# Unique collateral providing flow for an occluded iliac artery

**DOI:** 10.1016/j.jvscit.2021.05.002

**Published:** 2021-05-21

**Authors:** Anthony Feghali, Francesco Cardelli

**Affiliations:** Division of Vascular and Endovascular Surgery, SUNY Upstate Medical University, Syracuse, NY

**Figure:**
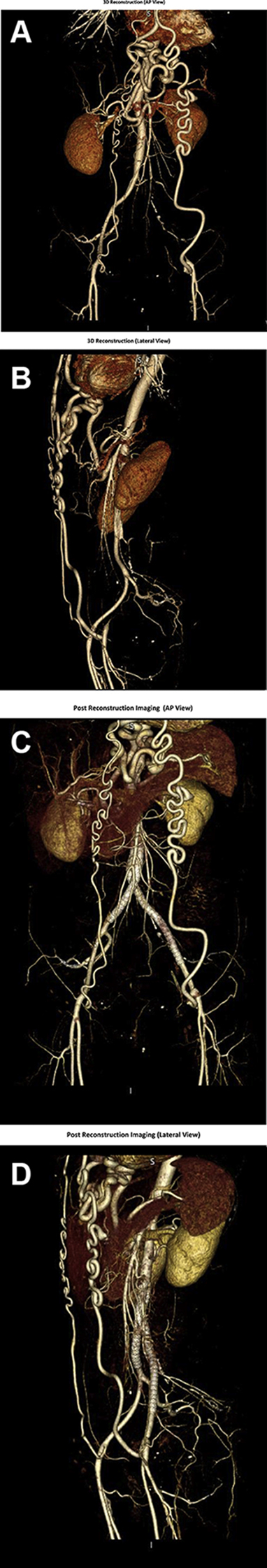

## Presentation of image

We present a 63-year-old female patient with a past medical history significant for previous smoking abuse, hypertension, and chronic pulmonary obstructive disease referred for worsening claudication. She had quit smoking 2 years prior and has had progressively worsening left lower extremity claudication with one to two blocks over the past 6 months. Her surgical history is significant for a cholecystectomy, c-section, and hysterectomy. On physical examination she had no palpable left femoral pulse and weak posterior tibial and dorsalis pedis pulses on doppler.

We obtained further imaging with a computed tomography angiography of the aorta with runoff to both lower extremities that showed a large and unique collateral. She was noted to have an occluded left iliac artery from the aorta to the femoral. Further, her infrarenal aorta showed approximately 50% stenosis/narrowing. More uniquely, the patient had reconstitution of blood flow from the left inferior epigastric vessel communicated with a replaced hepatic artery off the superior mesenteric artery proximally and left common femoral artery distally. Her celiac artery was significantly smaller in comparison with her superior mesenteric artery. Coming off her superior mesenteric artery was a replaced hepatic artery that branched and provided flow into both the left and right lobes of the liver. Beyond the liver, the replaced hepatic artery provided collateral flow into the inferior epigastric artery that reconstituted flow into the left femoral artery. Lastly, her inferior mesenteric artery was small but patent.

After discussion with the patient, we opted for surgical repair. The plan was to perform a covered endovascular reconstruction of the aortic bifurcation with femoral cutdown on the left femoral artery. If we could not cross the occluded vessel, then a right to left femoral bypass was considered. We were able to perform inline reconstruction with a covered endovascular reconstruction of the aortic bifurcation using Gore VBX stent grafts. Also, intraoperatively, we identified the large epigastric collateral and were able to preserve this vessel. Postoperatively, she did well and regained direct inline flow with palpable pulses. Follow-up imaging shows patency of the reconstruction with the patient reporting no further claudication.

## Discussion

The human body has the ability to create alternate pathways for distal perfusion when the native arteries become progressively more stenotic and eventually become occluded. This is most apparently seen during lower extremity angiograms, when multiple small collaterals can be observed filling with contrast in the setting of a stenotic or occluded femoral artery, for example. This process has been documented in patients with aortoiliac disease as well. A prospective study by Kim et al^1^ showed that of 15 patients with suspected or diagnosed chronic occlusive disease of the aorta or iliac arteries, all of them had developed a collateral pathway to reconstitute the external iliac artery, involving the internal thoracic artery, the superior epigastric artery, and the inferior epigastric artery. This parietal collateral pathway was present regardless of the level of aortic occlusion (thoracic vs abdominal) or etiology (atherosclerosis vs Takayasu's arteritis).

Collateral pathways can be categorized as systemic-systemic pathways (SSP) and visceral-visceral or visceral-systemic pathways (VSP), as reviewed in Hardman et al's pictorial essay published in September 2011.^2^ Embryologic development of branches stemming from the primitive dorsal aorta (SSP) as well as the ventral and lateral aorta (VSP) is ultimately what allows the developed arterial system to communicate and even fuse its branches and trunks, allowing for collateralization of flow. Mapping the arterial system by computed tomography arteriography with 3D reconstruction is an excellent tool to identify any collateral pathways in patients with significant aortoiliac disease or occlusion. Delineation of vascular anatomy in these patients is an essential component of preprocedural planning as a way to avoid compromising an already established inflow on which the patient may depend for distal perfusion. This is useful in both vascular surgical planning and planning of any other abdominal or cardiothoracic operations with the goal to preserve these pathways when present.

The body is remarkable in creating unique collaterals to adapt with vascular disease. We share this image to show the ability of the body to develop impressive collateral blood flow to compensate for vascular disease.
